# High Partial Auxeticity Induced by Nanochannels in [111]-Direction in a Simple Model with Yukawa Interactions

**DOI:** 10.3390/ma11122550

**Published:** 2018-12-14

**Authors:** Konstantin V. Tretiakov, Paweł M. Pigłowski, Jakub W. Narojczyk, Mikołaj Bilski, Krzysztof W. Wojciechowski

**Affiliations:** 1 Institute of Molecular Physics, Polish Academy of Sciences, Smoluchowskiego 17/19, 60-179 Poznan, Poland; pmp@ifmpan.poznan.pl (P.M.P.); narojczyk@ifmpan.poznan.pl (J.W.N.); kww@ifmpan.poznan.pl (K.W.W.); 2 Institute of Applied Mechanics, Poznań University of Technology, Jana Pawła II 24, 60–965 Poznań, Poland

**Keywords:** auxetics, negative Poisson’s ratio, nanochannel, colloidal crystal, model of nanocomposite

## Abstract

Computer simulations using Monte Carlo method in the isobaric-isothermal ensemble were used to investigate the impact of nanoinclusions in the form of very narrow channels in the [111]-direction on elastic properties of crystals, whose particles interact via Yukawa potential. The studies were performed for several selected values of Debye screening length (${\left(\kappa \sigma \right)}^{-1}$). It has been observed that introduction of the nanoinclusions into the system reduces the negative value of Poisson’s ratio towards $\left[110\right]\left[1\bar{1}0\right]$, maintaining practically constant values of Poisson’s ratio in the directions [100] and [111]. These studies also show that concentration of particles forming the nanoinclusions in the system has a significant effect on the value of Poisson’s ratio in the $\left[110\right]\left[1\bar{1}0\right]$-direction. A strong (more than fourfold) decrease of Poisson’s ratio in this direction was observed, from −0.147(3) (system without inclusions) to −0.614(14) (system with nanoinclusions) at κσ=10 when the inclusion particles constituted about 10 percent of all particles. The research also showed an increase in the degree of auxeticity in the system with increasing concentration of nanoinclusion particles for all the screening lengths considered.

## 1. Introduction

Composite materials are increasingly popular in today’s world mainly because of their various physical, mechanical and other properties, which are desirable in different applications [1]. Their unusual properties are used in electronics [2], in constructing the brakes of aircraft [3], or even as catalysts and adsorbents [4]. Recently, one can observe increase of interest in auxetic composites [5,6] and auxetic structures [7,8]. Auxetics, i.e., materials with negative Poisson’s ratio [9,10,11,12,13,14], exhibit an unusual mechanical property: they shrink transversally under compression and increase their transverse dimensions when stretched. This property encourages one to use these materials in various devices, e.g., molecular sieves, sensors, and other applications [15]. This makes them a subject of research in the context of looking for new auxetic materials and mechanisms leading to auxeticity at various scales—both microscopic [16,17,18,19,20,21,22] and macroscopic [23,24,25,26,27,28,29,30,31,32,33,34,35,36,37,38,39,40]. Recently, thin films of thickness in nanometer scale have been presented that exhibit zero Poisson’s ratio [41] and polymer nanocomposite foams that find applications for chemical sensors [42], electromagnetic shielding [43] or damping applications [44]. One of interesting ideas of looking for new auxetic materials is searching for auxetic composites at nano level, in particular molecular nanocomposites. This idea has been tested recently on models of colloidal crystals using computer simulations [45,46]. A reason for such studies was presented by Demirörs et al [47] who illustrated the possibility of producing single- and multicomponent colloidal arrays in complex three-dimensional structures. In this context, good systems to create models of nanocomposites are charge-stabilized colloidal crystals [48,49,50,51,52,53,54], in which particles interact through the hard-core repulsive Yukawa pair potential [48,49,50,52] and hard-sphere potential [55]. In such models, the hard-core repulsive Yukawa pair potential describes interactions between charged colloidal particles and interaction between nonionic colloids is modeled using the hard-sphere potential. In addition, particles interacting only with the hard potential are treated as inclusions into matrices of Yukawa particles interacting via the hard-core repulsive Yukawa pair potential. Elastic properties of both the hard-sphere crystals [56] and face-centered cubic (fcc) crystals of hard-core repulsive Yukawa particles [57] are well-known. Recently, auxetic properties of systems with inclusions in the form of nanochannels or nanolayers have been studied [45,46,58,59]. This research shows that the introduction of hard inclusions into crystal of Yukawa particles induces [46] or enhances [45,58,59] auxetic properties of nanocomposite models. As both hard-sphere and Yukawa crystals are anisotropic, orientation of inclusions is expected to be relevant for elastic properties of the resulting system.

The main goal of this work was to show a strong influence of the nanoinclusions on auxetic properties of the considered model materials. In particular, we demonstrated that the introduction of nanoinclusions in the form of nanochannels in the [111]-direction into crystal of Yukawa particles leads to high partial auxeticity of the system. The latter was shown to reduce more than four times the negative value of Poisson’s ratio in the $\left[110\right]\left[1\bar{1}0\right]$-direction. Moreover, the reduction of Poisson’s ratio in the $\left[110\right]\left[1\bar{1}0\right]$-direction occurred simultaneously with almost unchanging values of Poisson’s ratio in the other two main crystallographic directions, [100] and [111]. This gives a new possibility to achieve a huge enhancement of auxeticity in a selected direction.

## 2. Model, Method and Computation Details

### 2.1. The Model

**Figure 1:**
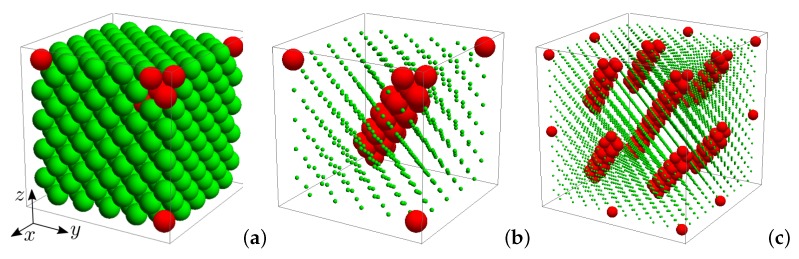
The structures of studied crystals with nanochannels in the [111]-crystallographic direction: (**a**) with green are marked particles which interact via the hard-core repulsive Yukawa pair potential (HCRYP), while red particles represent the hard spheres; (**b**) to better illustrate the structure of nanochannel, the centers of Yukawa particles are marked by green dots; and (**c**) periodic boundary conditions result in the structure with periodic array of nanochannels.

We considered a system consisting of $N=N_{Y}+N_{HS}$ particles in periodic boundary conditions that initially form a face-centered cubic structure. The model system is shown in Figure 1. Particles $N_{Y}$, marked in figure with green, interact mutually through the hard-core repulsive Yukawa pair potential (HCRYP) [48,49,50,52]:

(1) $$\beta u_{ij}=\left\{\begin{array}{ccc} \infty & , & r_{ij}<\sigma \\ \beta \epsilon \frac{\exp[-\kappa \sigma \left(r_{ij}/\sigma -1\right)]}{r_{ij}/\sigma } & , & r_{ij}\geq \sigma . \end{array}\right.$$

where $\beta =1/(k_{B}T)$, $k_{B}$ is the Boltzmann constant, *T* is the temperature, σ is the diameter of the particle’s hard core, ϵ is the contact potential, $\kappa^{-1}$ is the Debye screening length and $r_{ij}$ is the distance between the centers of *i* and *j* particles. On the other hand, the particles $N_{HS}$ marked with red in Figure 1 represent the nanoinclusions in the form of a nanochannel in the crystallographic direction [111], which interact with each other and with the Yukawa particles via the hard sphere potential (HSP):

(2) $$\beta u_{ij}=\left\{\begin{array}{ccc} \infty & , & r_{ij}<\sigma ,\\ 0 & , & r_{ij}\geq \sigma . \end{array}\right.$$

As a result of the introduction of a nanochannel, we have a situation in which particles in the crystal can interact with different potentials (hard or Yukawa potential), which can lead to changes in interparticle distances and in crystallographic symmetry of the studied system. In this work, we considered a system with a nanochannel oriented along [111] direction, whose particles are contained in a cylinder of a diameter $\sigma /\sqrt{3}\leq d<\sigma$. Application of periodic boundary conditions results in a system consisting of infinitely many parallel nanochannels (Figure 1c). Similar to previous works [45,46], to analyze the impact of the nanochannels on elastic properties of the studied systems, we introduced the “concentration” parameter describing the ratio of the number of hard spheres forming the channel ($N_{HS}$) to all particles in the system:

(3) $$c=\frac{N_{HS}}{N}\times 100\%.$$

In Table 1, concentrations of the nanoinclusion particles and parameters of the systems studied in this work are given. All symbols and signs used in the paper are explained in the text of the paper. Additional symbols useful to understand the additional notions are collected in the Supplementary Materials.

### 2.2. Method

The studies were conducted by computer simulations using Monte Carlo (MC) method in an isobaric-isothermal (NpT) ensemble. To determine elastic properties of the studied systems, the Parrinello–Rahman method [60,61,62,63] with variable shape of the periodic box was used (details in the Supplementary Materials). According to this approach, tensor of elastic compliances $S_{ijkl}$ can be determined directly by analyzing the fluctuations of the strain tensor:

(4) $$S_{ijkl}=\langle ∆\varepsilon_{ij}∆\varepsilon_{kl}\rangle V_{p}/k_{B}T\;,$$

where $\varepsilon_{ij}$ are elements of the strain tensor $∆\varepsilon_{ij}\equiv \varepsilon_{ij}-\langle \varepsilon_{ij}\rangle$, $V_{p}$ is the equilibrium volume of the system at pressure *P*, and

(5) $$\varepsilon =\left(h_{0}^{-1}\cdot h\cdot h\cdot h_{0}^{-1}-I\right)/2\;.$$

In Equation (5), $h_{0}\equiv \langle h\rangle$ is the reference box matrix; h matrix, in which the columns are formed by the edges of the simulation box, represents instantaneous values of the simulation box in each simulation step; and I is the identity matrix. $\langle \ldots \rangle$ means the average over the NpT ensemble (matrices $h_{0}$ and h are kept symmetric). Knowledge of all (21 in the general case) elements of the elastic compliance tensor allows for a full description of elastic properties of the studied system and for determination of Poisson’s ratio in any crystallographic direction [46,64]:

(6) $$\nu_{nm}=-\frac{m_{i}m_{j}S_{ijkl}n_{k}n_{l}}{n_{p}n_{r}S_{prst}n_{s}n_{t}}\;,$$

where: $n_{i}$ and $m_{i}$ are the components of unit vectors $\vec{n}$ and $\vec{m}$, respectively. The $\vec{n}$ versor represents the direction in which the external stress is applied. The $\vec{m}$ versor indicates the direction in which one measures the deformation induced by applied stress (see the Supplementary Materials).

The possibility of determining Poisson’s ratio in any crystallographic direction allows one to analyze the impact of the nanochannel on auxetic properties in the entire system, not only in a single specific direction. For this purpose, we calculated the coefficient, previously proposed by us [65], which determines the degree of auxeticity of the system:

(7) $$\chi =\sqrt[{3}]{\frac{3A}{4\pi }}\;,$$

where

(8) $$A=\int_{0}^{2\pi }\;\;\int_{0}^{\pi }\int_{0}^{R(\theta ,\phi )}r^{2}dr\sin\theta d\theta d\phi \;,$$

and

(9) $$R\left(\theta ,\phi \right)=\frac{1}{2\pi }\int_{0}^{\pi }\left(\left|\nu_{n(\theta ,\phi )}\left(\alpha \right)\right|-\nu_{n(\theta ,\phi )}\left(\alpha \right)\right)d\alpha$$

is the average *negative* value of Poisson’s ratio towards $\vec{n}$-direction, while α is the angle between the $\vec{m}$-direction, in which the Poisson’s ratio is measured, and the direction resulting from intersection of the OXY plane and the plane perpendicular to $\vec{n}$ [45]. The derivation of χ is described and illustrated in detail in the Supplementary Materials of the work [65].

### 2.3. Computational Details

Based on the pre-determined phase diagrams [48,50] and on the results of elastic properties of Yukawa crystals [57] and hard spheres [56], simulations were conducted at reduced pressure $p^{*}\equiv \beta P\sigma^{3}=100$ for contact potential values βϵ=20 and three Debye screening lengths ${\left(\kappa \sigma \right)}^{-1}$. The length of a single simulation run was $2\times 10^{6}$ MC cycles after equilibration (the MC cycle contains a trial step performed for each particle, which results in N single particle trial steps, and integer part of the square root of *N* trail steps in which the periodic box matrix is changed (see, e.g., [63])). Simulations for each of the phase points studied were performed for at least 10 independent structures, for which the average values of elastic compliances were determined.

The intermolecular potential (Equation (1)) was truncated at a distance $r_{\mathrm{cut}}=2.5\sigma$. Therefore, long-range corrections were considered during calculations of potential energy of the system. The choice of $r_{\mathrm{cut}}=2.5\sigma$ resulted from the fact that intermolecular potentials for parameters considered in this work are short-ranged [65]. The correctness of selection of the value $r_{\mathrm{cut}}$ was verified by calculating Poisson’s ratio in the auxetic direction ($\left[110\right]\left[1\bar{1}0\right]$) with respect to $r_{\mathrm{cut}}$. It was found that the results obtained for $r_{\mathrm{cut}}=2.5\sigma$ are in very good agreement with results obtained for larger distances of potential truncation [65].

## 3. Results and Discussion

### 3.1. Elastic Compliances and Symmetry of the System

**Figure 2:**
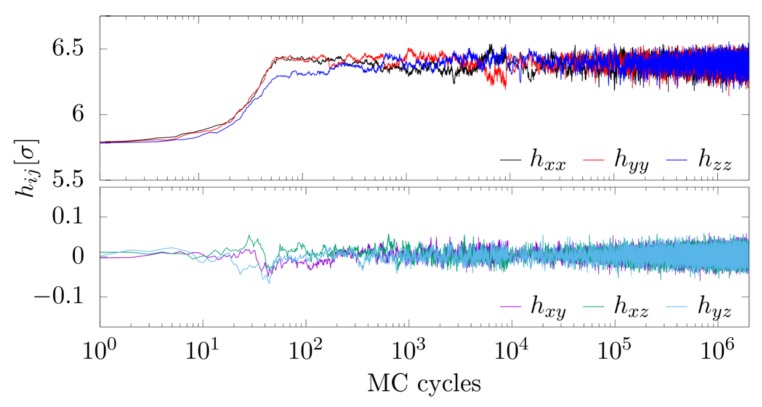
Components of the simulation box of the system versus MC cycles, studied at: N=256, c=10.94%, $p^{*}=100$, κσ=10 and βϵ=20.

As mentioned above, initial structure of the system was the fcc structure. Introduction of nanoinclusions into the system changed its structure, which during the simulation was manifested by the change of the form of the box matrix. As shown in Figure 2, the equilibrium form of the box matrix was achieved after about $10^{3}$ MC cycles. The matrix is symmetrical and describes the shape of the rhombohedron:

(10) $$\langle h\rangle =\left[\begin{array}{ccc} \langle h_{xx}\rangle & \langle h_{xy}\rangle & \langle h_{xy}\rangle \\ \cdot & \langle h_{xx}\rangle & \langle h_{xy}\rangle \\ \cdot & \cdot & \langle h_{xx}\rangle \end{array}\right]\;.$$

As a result of the simulations performed, using Equations (4) and (5), elastic compliance tensor S was determined, which using the Voigt (matrix) notation can be written in the following form:

(11) $$\left[\begin{array}{cccccc} S_{11} & S_{12} & S_{12} & S_{14} & S_{15} & S_{15}\\ \cdot & S_{11} & S_{12} & S_{15} & S_{14} & S_{15}\\ \cdot & \cdot & S_{11} & S_{15} & S_{15} & S_{14}\\ \cdot & \cdot & \cdot & S_{44} & S_{45} & S_{45}\\ \cdot & \cdot & \cdot & \cdot & S_{44} & S_{45}\\ \cdot & \cdot & \cdot & \cdot & \cdot & S_{44} \end{array}\right]\;.$$

To simplify the form of the compliance tensor, the coordinate system was rotated in such a way that the [111]-direction became the [001]-direction. In the new coordinate system, the compliance tensor reads $S_{ijkl}^{\prime }=R_{ip}R_{jr}R_{ks}R_{lt}S_{prst}$, where $R_{ij}$ represents the components of the rotation matrix in the form:

(12) $$R=\frac{1}{\sqrt{6}}\left[\begin{array}{ccc} -\sqrt{3} & \sqrt{3} & 0\\ -1 & -1 & 2\\ \sqrt{2} & \sqrt{2} & \sqrt{2} \end{array}\right]\;.$$

Finally, the $S_{ijkl}^{\prime }$ tensor can be written as a matrix $S_{\alpha \beta }^{\prime }$ using the Voigt notation [66]:

(13) $$S^{\prime }=\left[\begin{array}{cccccc} S_{11}^{\prime } & S_{12}^{\prime } & S_{13}^{\prime } & S_{14}^{\prime } & 0 & 0\\ \cdot & S_{11}^{\prime } & S_{13}^{\prime } & -S_{14}^{\prime } & 0 & 0\\ \cdot & \cdot & S_{33}^{\prime } & 0 & 0 & 0\\ \cdot & \cdot & \cdot & S_{44}^{\prime } & 0 & 0\\ \cdot & \cdot & \cdot & \cdot & S_{44}^{\prime } & 2S_{14}^{\prime }\\ \cdot & \cdot & \cdot & \cdot & \cdot & 2(S_{11}^{\prime }-S_{12}^{\prime }) \end{array}\right]\;.$$

Relations between the matrix elements in Equations (11) and (13) are as follows:

$$\begin{array}{ccc} S_{11}^{\prime } & = & \frac{1}{4}\left(2S_{11}+2S_{12}-4S_{15}+S_{44}\right),\\ S_{33}^{\prime } & = & \frac{1}{3}\left(S_{11}+2S_{12}+2S_{14}+4S_{15}+S_{44}+2S_{45}\right),\\ S_{12}^{\prime } & = & \frac{1}{12}\left(2S_{11}+10S_{12}-8S_{14}-4S_{15}-S_{44}+4S_{45}\right),\\ S_{13}^{\prime } & = & \frac{1}{6}\left(2S_{11}+4S_{12}+S_{14}+2S_{15}-S_{44}-2S_{45}\right),\\ S_{44}^{\prime } & = & \frac{1}{3}\left(4S_{11}-4S_{12}-4S_{14}+4S_{15}+S_{44}-S_{45}\right),\\ S_{14}^{\prime } & = & \frac{1}{3\sqrt{2}}\left(-2S_{11}+2S_{12}-S_{14}+S_{15}+S_{44}-S_{45}\right). \end{array}$$

**Figure 3:**
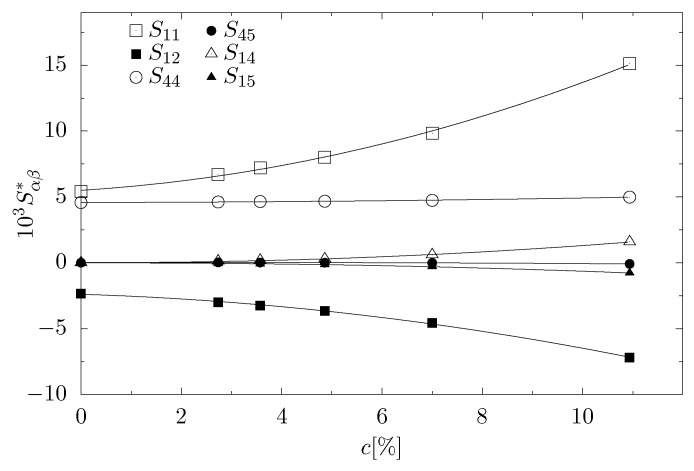
Elements of the elastic compliance matrix S with respect to the concentration, studied at: $p^{*}=100$, κσ=10 and βϵ=20.

The form of the elastic compliance matrix in Equation (13) indicates that the system has a trigonal symmetry [66]. Thus, the presence of the nanoinclusions in the form of a channel in the [111]-crystallographic direction resulted in a change of the crystallographic structure from the fcc to trigonal. Increasing concentration of particles forming the nanoinclusion had a strong impact on elastic properties of the studied system, as shown in Figure 3. The values of $S_{11}$ and $S_{12}$ changed significantly with the increase of *c*; an increase of $S_{11}$ and a decrease of $S_{12}$ were observed. In addition, elements $S_{14}$, $S_{15}$ and $S_{45}$ became non-zero, with component $S_{45}$ taking negative values, very close to 0. These changes have a significant impact on Poisson’s ratio.

### 3.2. Size Effects

**Figure 4:**
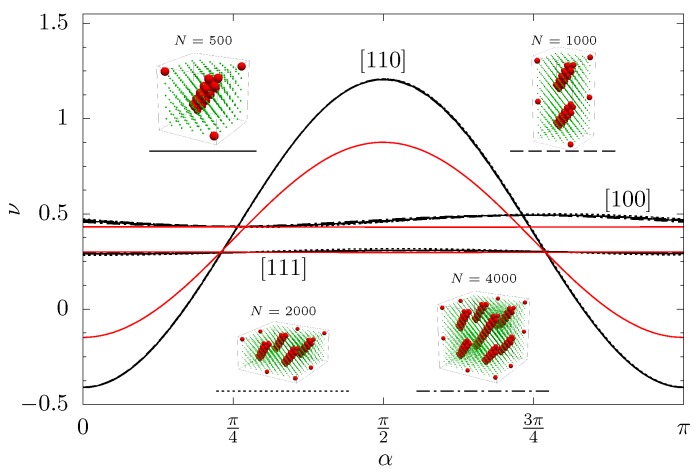
Poisson’s ratio in three crystallographic directions for various size of the system (*N* = 500, 1000, 2000, and 4000) as a function of the angle α (with c=7%, $p^{*}=100$, κσ=10 and βϵ=20). Red lines represent Poisson’s ratio in the respective crystallographic directions of the system without nanoinclusions.

Influence of the number of supercells and the shape of the simulated periodic box on Poisson’s ratio was analyzed (see Figure 4). A weak dependence of Poisson’s ratio with respect to the number and distribution of the supercells in the periodic box was observed, similar to previous studies [45] regarding the model with channel towards [001]. Thus, in this paper, the simulation results are presented for systems represented by single elementary supercells, whose parameters are given in Table 1.

In Figure 4, it is worth noting the small changes of Poisson’s ratio in the crystallographic directions [100] and [111], which were related to the presence of the nanoinclusions. On the other hand, in the [110]-direction, large changes of Poisson’s ratio in comparison to the system without the nanoinclusions were observed.

### 3.3. Poisson’s Ratio

**Figure 5:**
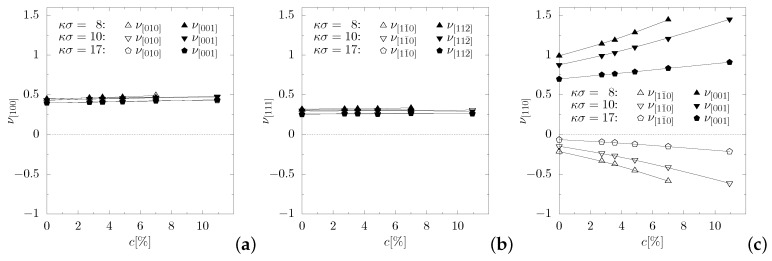
Poisson’s ratio of Yukawa crystals with nanochannels in the main crystallographic directions with respect to the concentration: (**a**) [100]; (**b**) [111]; and (**c**) [110].

In general, Poisson’s ratio is influenced by both the concentration of particles forming the inclusion (*c*) and the value of the screening length of the potential (${\left(\kappa \sigma \right)}^{-1}$). In Figure 5, Poisson’s ratios in the main crystallographic directions are plotted for studied values of screening length, with respect to the concentration. Figure 5a,b shows a rather weak influence of presence of the nanoinclusions on Poisson’s ratio in the directions [100] and [111], as well as its poor dependence on the screening length—which is consistent with previous studies [57]. In the direction $\left[110\right]\left[1\bar{1}0\right]$ (Figure 5c), for each screening length studied, the increase in concentration caused Poisson’s ratio to decrease in direction $\left[110\right]\left[1\bar{1}0\right]$, i.e., enhancing auxetic properties. The lowest value of Poisson’s ratio observed in these studies equals $\nu_{\min}=-0.614\left(14\right)$ and was measured in the $\left[110\right]\left[1\bar{1}0\right]$-direction for concentration c=10.9375%, κσ=10 and βϵ=20. The analysis of these dependencies in the main crystallographic directions suggests the possibility of selective strengthening of auxetic properties of the system. Namely, one can lower Poisson’s ratio for larger concentrations of particles of nanoinclusions in one of the main crystallographic directions ($\left[110\right]\left[1\bar{1}0\right]$), while, in the crystallographic directions [100] and [111], the value of Poisson’s ratio remains almost constant (Figure 5).

**Figure 6:**
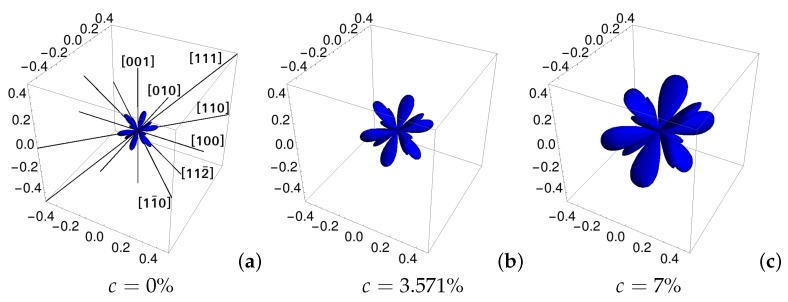
Absolute value of the minimal negative Poisson’s ratio in all crystallographic directions for Yukawa systems with nanochannels in the [111]-direction for inverse screening length κσ=10 and various concentrations: (**a**) c=0%; (**b**) c=3.571%; and (**c**) c=7%.

On the other hand, the minimum values of Poisson’s ratio determined in all the auxetic crystallographic directions (Figure 6) strongly decreased with the increase of concentration of particles forming nanoinclusions. This observation indicates the enhancement of auxetic properties in the whole system for higher values of concentration of particles of nanoinclusions.

### 3.4. The Degree of Auxeticity

**Figure 7:**
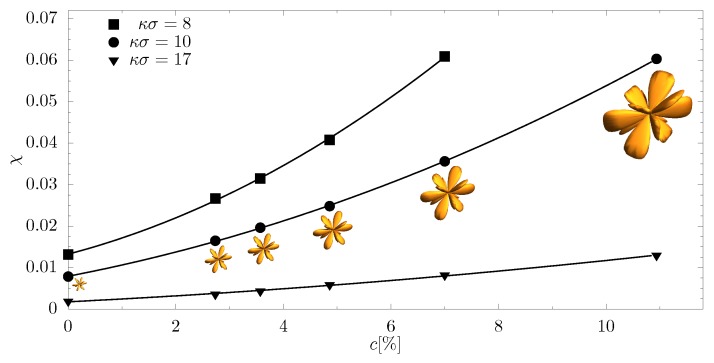
Degree of auxeticity with respect to the concentration for various values of screening length. The inserts in the figure show the average absolute value of negative Poisson’s ratio (Equation (9)) in all crystallographic directions for κσ=10. The surface consists of the points that represent R(θ,ϕ). All the inserted figures are presented at the same scale.

To determine the influence of nanoinclusions on the auxetic properties of the system, dependence of the degree of auxeticity χ (Equation (7)) with respect to the concentration of nanoinclusion particles was determined (Figure 7). The inserts in Figure 7 show the surface of the average negative values of Poisson’s ratio (Equation (9)) in all crystallographic directions for κσ=10. The increase of volume of the drawings clearly signifies the strengthening of auxetic properties and translates directly into the growth of χ with concentration. The resulting value of χ=0.061 is one of the largest of all Yukawa systems with structural modifications studied thus far [45,46,58,65]. Analysis of the results obtained indicates a significant increase of the degree of auxeticity of the whole system: 4.5 times for κσ=8, 7.5 times for κσ=10 and almost 7 times for κσ=17. Based on these observations, we can postulate a high partial auxeticity in crystals with Yukawa interactions induced by nanoinclusions in the form of nanochannels in the [111]-direction.

## 4. Conclusions

Using the Monte Carlo method, computer simulations of model crystals, in which particles interact with each other by Yukawa potential and the particles constituting the inclusions interact with all other particles by hard potential, were performed. The simulations showed that introduction of inclusions into the model system in the form of nanochannels in the crystallographic direction [111] causes a change of its crystal structure from the fcc to a trigonal one. Elastic properties of model crystals with various concentrations of nanoinclusion particles were determined for several values of Debye screening length. These calculations showed a significant reduction of Poisson’s ratio in the $\left[110\right]\left[1\bar{1}0\right]$-direction with increasing concentration of particles forming the channels. The value of Poisson’s ratio in this direction decreased more than four times from −0.147(3) to −0.614(14). A separate consideration was given to the fact that the presence of nanoinclusions in a system, which reduced the negative value of Poisson’s ratio in the $\left[110\right]\left[1\bar{1}0\right]$-direction, almost did not affect values of Poisson’s ratio in the other two main crystallographic directions, [100] and [111]. In these directions, Poisson’s ratio has values close to those in the system without inclusions. It is worth noting that this effect was observed for all studied values of the Debye screening length.

Finally, one should stress that the model crystals with nanoinclusions in the form of channels in the crystallographic direction [111] show the highest degree of auxeticity among all systems of this type studied thus far. It was observed that the presence of nanoinclusions results in a significant increase of the degree of auxeticity of the whole system: 4.5 times for κσ=8, 7.5 times for κσ=10 and almost 7 times for κσ=17.

One should realize that the model under consideration is based on certain simplifications and assumptions, inter alia related to the interaction potentials of colloidal particles and the possibility of modifying crystalline structure. However, we believe that the rapid development of nanotechnology (in particular, the work by Demirörs et al. [47]) brings us closer to obtaining real systems similar to the models presented in this paper. Then, one can speculate that materials based on the considered model could be used, e.g., as sensors or damping applications for vibration control in a given direction. Besides, the results obtained in this work have a general character and can be helpful in construction of materials with given elastic properties and not only in the nanoscale. Undoubtedly, an important aspect of this research is also the indication of the direction of research on the synthesis of auxetic nanocomposites.

## Figures and Tables

**Table 1:** Summary of the parameters of the studied systems: *n*, number of fcc cells on the edge of the system; *N*, total number of particles in the system; $N_{HS}$, number of hard spheres forming the inclusions; *c*, concentration of nanochannel particles.

*n*	$N=4n^{3}$	*N_HS_* = 7n	*c*
4	256	28	10.94%
5	500	35	7.000%
6	864	42	4.861%
7	1372	49	3.571%
8	2048	56	2.734%

## Supplementary Materials

The following are available online at http://www.mdpi.com/1996-1944/11/12/2550/s1.

## Abbreviations

The following abbreviations are used in this manuscript:

MC	Monte Carlo
HS	Hard Sphere
HCRYP	Hard-Core Repulsive Yukawa Potential
