# Supplementary material for: High Partial Auxeticity Induced by Nanochannels in [111]-Direction in a Simple Model with Yukawa Interactions
Source: Materials (Basel). 2018 Dec 14;11(12):2550. doi: 10.3390/ma11122550 (PMC6315580; doi:10.3390/ma11122550)
Supplement: Supplementary file 1 [file materials-11-02550-s001.pdf]

materials

MDPI

*Article*

# Supplementary Information for ”High partial auxeticity in simple model with Yukawa interactions induced by nanochannels in [111]-direction”

**Konstantin V. Tretiakov[1], Paweł M. Pigłowski[1], Jakub W. Narojczyk[1], Mikołaj Bilski[2] and Krzysztof W. Wojciechowski[1]**

1 Institute of Molecular Physics, Polish Academy of Sciences, M. Smoluchowskiego 17, 60-179 Poznań, Poland
2 Institute of Applied Mechanics, Poznań University of Technology, Jana Pawła II 24, 60–965 Poznań, Poland
* tretiakov@ifmpan.poznan.pl, Tel.: +48-61-689-52-76; mikolaj.bilski@put.poznan.pl

Published: date

## 1. Used nomenclature

The signs used in the manuscript are shown below

- $N$ – the number of particles
- $N_{HS}$ – the number of particles forming inclusion
- $N_Y$ – the number of ’Yukawa’ particles
- $n$ – the number of fcc cells on the edge of the system
- $c$ – the concentration of the nanoinclusion particles
- $\sigma$ – the particles’ diameter
- $\kappa^{-1}$ – the Debye’s screening length
- $\epsilon$ – the contact potential
- $\beta = 1/(k_B T)$
- $k_B$ – the Boltzmann constant
- $T$ – the temperature
- $r_{ij}$ – distance between $i$-th and $j$-th particle
- $S_{ijkl}$ – component of elastic compliance tensor
- $\varepsilon_{ij}$ – component of strain tensor
- $V_p$ – equilibrium volume of the system
- $P$ – pressure
- $p^* \equiv \beta P \sigma^3$ – reduced pressure
- $\mathbf{h}$ – the box matrix
- $\mathbf{h}_0 \equiv \langle \mathbf{h} \rangle$ – the reference box matrix
- $I$ – identity matrix
- $\delta_{ij}$ – the Kronecker delta
- $n_i$ – the $i$-component of a unit vector in the direction of the applied stress
- $m_i$ – the $i$-component of a unit vector in the direction in which the reaction of the system is observed.
- $\nu_{nm}$ – the Poisson’s ratio
- $\chi$ – the degree of auxeticity

In this paper Voigt’s notation and Einstein’s summation are used.

## 2. Computations of the elastic compliances

The Lagrangian strain tensor can be expressed as [1]:

$$\varepsilon_{ij} \equiv \left( \partial_i u_j + \partial_j u_i + \sum_k \partial_i u_k \partial_j u_k \right) /2\,, \tag{1}$$

where $u_i \equiv x_i - X_i$ is the displacement vector and $X_i$, $x_i$ describe respectively the undeformed state and the state under the deformation [1]. Under constant isotropic pressure ($P$) the expansion of the change of free enthalpy (Gibbs free energy), $\Delta G$, caused by deformation of a crystal has the form [2]:

$$\Delta G = \frac{1}{2} V_p B_{ijkl} \varepsilon_{ij} \varepsilon_{kl} + ... \tag{2}$$

where $B_{ijkl}$ are the components of the elastic constants tensor at fixed temperature and pressure $P$ (the Einstein's summations is used). Under the isotropic pressure conditions, $\sigma_{ij} \equiv -P\delta_{ij}$, the elastic constans $B_{ijkl}$ form the relation between the components of the strain tensor $\varepsilon_{kl}$ and the stress tensor $\sigma_{ij}$ [3] (the Hooke's law):

$$\Delta\sigma_{ij} = B_{ijkl}\varepsilon_{kl}, \tag{3}$$

where $\Delta\sigma_{ij} \equiv \sigma_{ij} + P\delta_{ij}$. By inversion, the above reads:

$$\varepsilon_{ij} = S_{ijkl}\Delta\sigma_{kl}, \tag{4}$$

where $S_{ijkl}$ is the elastic compliance tensor, a fourth-rank tensor which remains unchanged when replacing $i$-$j$, $k$-$l$ and $ij$-$kl$. The elastic compliances are related to the elastic constants tensor elements by the following equality [4]:

$$S_{iklm} B_{lmpq} = \frac{1}{2} \left( \delta_{ip}\delta_{kq} + \delta_{iq}\delta_{kp} \right) . \tag{5}$$

In computer simulations the strain tensor is obtained from two matrices - the **h** matrix describing the system's state (under pressure $P$) and reference box matrix [5,6] $\mathbf{h}_0$ ($\mathbf{h}_0 \equiv \langle \mathbf{h} \rangle$):

$$'' = \frac{1}{2} \left( \mathbf{h}_0^{-1}.\mathbf{h}.\mathbf{h}.\mathbf{h}_0^{-1} - \mathbf{I} \right) , \tag{6}$$

where $\mathbf{I}$ is the unit matrix of the dimensionality 3. Both $\mathbf{h}$ and $\mathbf{h}_0$ are kept symmetric during simulations. Considering that at equilibrium $\varepsilon_{ij} = 0$, it has been shown [5] that fluctuations of $\varepsilon_{ij}$ are related to the elastic compliance tensor $S_{ijkl}$:

$$S_{ijkl} = \langle \Delta\varepsilon_{ij} \Delta\varepsilon_{kl} \rangle \frac{V_p}{k_B T}, \tag{7}$$

where $\Delta\varepsilon_{ij}$ is the difference between reference and instantaneous states, and the $\langle ... \rangle$ denotes the averaging in the isothermal–isobaric ensemble:

$$\langle f \rangle = \frac{\int d\varepsilon^{(6)} f \exp(-G/k_B T)}{\int d\varepsilon^{(6)} \exp(-G/k_B T)} \tag{8}$$

(for more details see [3,7,8]).

## 3. $\vec{n}$ and $\vec{m}$ directions

Based on the knowledge of the full tensor of elastic compliances one can calculate the Poisson's ratio for arbitrary direction [9]

$$\nu_{nm} = -\frac{m_i m_j S_{ijkl} n_k n_l}{n_p n_r S_{prst} n_s n_t}\,, \tag{9}$$

In the equation (9) $\vec{\mathbf{n}}$ and $\vec{\mathbf{m}}$ are unit vectors indicating selected pair of directions (illustrated in the Figure 1) for which the Poisson's ratio is calculated. The $\vec{\mathbf{n}} = (n_x, n_y, n_z)$ vector is oriented in the direction of the applied stress (according to the definition of the Poisson's ratio). The $\vec{\mathbf{m}}$ represents the direction in which the reaction of the system on the applied stress is observed. It is located on the plane orthogonal to $\vec{\mathbf{n}}$, spanned by vectors $\vec{\mathbf{m}}_1$ and $\vec{\mathbf{m}}_2$:

$$\hat{\mathbf{m}}_1 = \frac{\hat{\mathbf{k}} \times \hat{\mathbf{n}}}{\sqrt{(\hat{\mathbf{k}} \times \hat{\mathbf{n}}) \cdot (\hat{\mathbf{k}} \times \hat{\mathbf{n}})}} = \frac{1}{\sqrt{n_x^2 + n_y^2}} \left(-n_y, n_x, 0\right), \tag{10}$$

$$\hat{\mathbf{m}}_2 = \hat{\mathbf{n}} \times \hat{\mathbf{m}}_1 = \frac{1}{\sqrt{n_x^2 + n_y^2}} \left(-n_x n_z, -n_y n_z, n_x^2 + n_y^2\right), \tag{11}$$

where $\hat{\mathbf{k}}$ is the versor of the $Oz$ axis. The versor is the unit vector denoted by symbol $\hat{}$. The $\alpha$ angle describes the orientation of $\vec{\mathbf{m}}$ vector on that plane:

$$\vec{\mathbf{m}} = \hat{\mathbf{m}}_1 \cos\alpha + \hat{\mathbf{m}}_2 \sin\alpha\ . \tag{12}$$

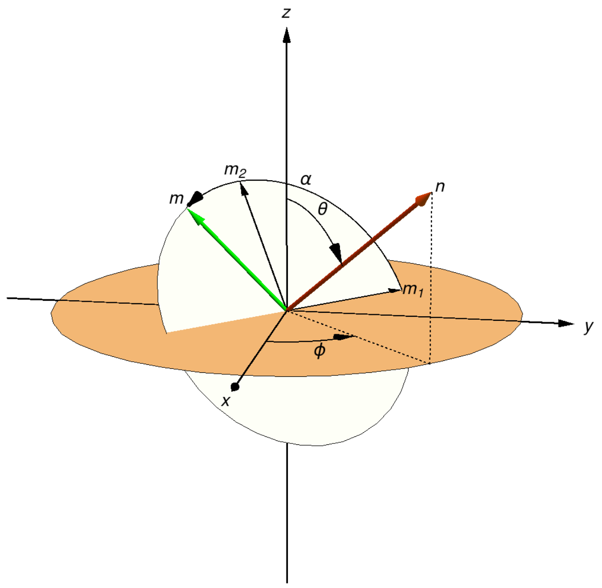


**Figure 1.** Spherical coordinates: $\vec{\mathbf{n}}$ (described by polar and azimuthal angles $\theta$, $\phi$) and $\vec{\mathbf{m}}$ (described by $\alpha$ angle). $\alpha$ is the angle between $\vec{\mathbf{m}}$ and $\hat{\mathbf{m}}_1$ ($\hat{\mathbf{m}}_1$ is the versor created by plane $Oxy$ and plane orthogonal to $\vec{\mathbf{n}}$).

## References

1. L. D. Landau and E. M. Lifshitz. *Theory of Elasticity*. Pergamon Press, London, 1986.
2. K. W. Wojciechowski. Negative Poisson ratios at negative pressures. *Molecular Physics Reports*, 10: pp. 129–136, 1995.
3. K. W. Wojciechowski, K. V. Tretiakov, and M. Kowalik. Elastic properties of dense solid phases of hard cyclic pentamers and heptamers in two dimensions. *Phys. Rev. E*, 67: 036121, 2003.
4. J. H. Weiner. *Statistical Mechanics of Elasticity*. Wiley, New York, 1983.
5. M. Parrinello and A. Rahman. Polymorphic transitions in single crystals: A new molecular dynamics method. *J. Appl. Phys.*, 52: pp. 7182–7190, 1981.
6. M. Parrinello and A. Rahman. Strain fluctuations and elastic constants. *J. Chem. Phys.*, 76: pp. 2662–2666, 1982.
7. K. W. Wojciechowski, K. V. Tretiakov, A. C. Brańka, and M. Kowalik. Elastic properties of two-dimensional hard disks in the close-packing limit. *J. Chem. Phys.*, 119: 939, 2003.

8. K. W. Wojciechowski. Computer simulations of elastic constants without calculating derivatives of the interaction potential. *Comp. Meth. Sci. Technol.*, 8(2): pp. 77–83, 2003.
9. S. P. Tokmakova. Stereographic projections of Poisson's ratio in auxetic crystals. *Phys. Status Solidi B-Basic Solid State Phys.*, 242(3): pp. 721–729, 2005.

© 2018 by the authors. Licensee MDPI, Basel, Switzerland. This article is an open access article distributed under the terms and conditions of the Creative Commons Attribution (CC BY) license (http://creativecommons.org/licenses/by/4.0/).
